# Sphingosine Kinase: A Novel Putative Target for the Prevention of Infection-Triggered Preterm Birth

**DOI:** 10.1155/2013/302952

**Published:** 2013-05-26

**Authors:** Vibhuti Vyas, Charles R. Ashby, Sandra E. Reznik

**Affiliations:** ^1^Department of Pharmaceutical Sciences, College of Pharmacy and Health Sciences, St. John's University, Queens, NY 11439, USA; ^2^Departments of Pathology, Obstetrics, Gynecology, and Women's Health, Montefiore Medical Center, Albert Einstein College of Medicine, Bronx, NY 10467, USA

## Abstract

Preterm birth is defined as any delivery before 37 complete weeks of gestation. It is a universal challenge in the field of obstetrics owing to its high rate of mortality, long-term morbidity, associated human suffering and economic burden. In the United States, about 12.18% deliveries in 2009 were preterm, producing an exorbitant cost of $5.8 billion. Infection-associated premature rupture of membranes (PROM) accounts for 40% of extremely preterm births (<28 weeks of gestation). Major research efforts are directed towards improving the understanding of the pathophysiology of preterm birth and ways to prevent or at least postpone delivery. Endothelin-1 (ET-1) is a potent vasoconstrictor that plays a significant role in infection-triggered preterm birth. Its involvement in a number of pathological mechanisms and its elevation in preterm delivered amniotic fluid samples implicate it in preterm birth. Sphingosine kinase (SphK) is a ubiquitous enzyme responsible for the production of sphingosine-1-phosphate (S1P). S1P acts as second messenger in a number of cell proliferation and survival pathways. SphK is found to play a key role in ET-1 mediated myometrial contraction. This review highlights SphK as a prospective target with great potential to prevent preterm birth.

## 1. Introduction

Preterm birth is a global challenge in obstetrics accounting for most long-term disabilities and mortalities in neonates and a significant economic burden to society [1]. The World Health Organization (WHO) defines preterm birth as delivery before the completion of 37 weeks of gestation [2]. Preterm delivery includes spontaneous preterm births as well as deliveries performed by clinical providers to avoid unfavorable sequelae for the mother or fetus. Blencowe et al. [3], in The Lancet, presents worldwide, regional, and national preterm birth data from 184 countries in 2010. Their studies estimate 14.9 million babies born preterm, which comprise 11.1% of all live births worldwide. The US ranks among the ten countries that have the highest rates of preterm births [3]. The US preterm birth rate has increased significantly since 1990 with an all time high of 12.33% in 2008 [4–7]. The rate of late preterm births (at 34–36 weeks) decreased from 8.77% to 8.66% between 2008 and 2009, whereas the early preterm rate (<34 weeks) decreased from 3.56% to 3.51% [4]. Preterm births cost the US a sum of $5.8 billion annually for the hospitalization of preterm infants/low birth weight infants. The average cost for intensive care of an extremely preterm infant (<28 weeks of gestation) is $65,600 [8, 9]. The real challenge lies in taking care of an extremely preterm infant. The advances in neonatology have improved the survival rates of extremely premature and extremely small infants. Larroque et al. [10] reported 78% survival in infants born at 28 weeks and 97% survival at 32 weeks. Lorenz and colleagues [11] have studied the effect of prematurity on the mortality and developmental disability of extremely immature (EI) (born <26 weeks of gestation) and extremely small (ES) (weighing <1000 g at birth) infants. They studied major neurodevelopment disabilities among infants due to preterm delivery. Their results suggest that 14% of EI and ES premature infants suffer from mental retardation, cerebral palsy is observed in 12% of EI survivors and 8% of ES survivors, blindness is found in 8% of EI and ES survivors, and 3% of the EI and ES population suffer from deafness [11]. Lorenz et al. thus concluded “Increasing survival of these infants has resulted in a steadily increasing prevalence of children with disabilities.” This situation requires us to ameliorate our knowledge about the pathology of preterm delivery.

Preterm birth is a complicated phenotype presenting a diversity of etiologic, biochemical, and genetic factors making it clinically difficult to understand [12]. Preterm birth ramifies into three clinical forms: spontaneous preterm labor (40%), premature rupture of membranes (40%), and fetal-maternal complications (20%) [13–15]. The study of patterns, etiologies, and occurrence of preterm delivery in defined populations has revealed the following risk factors: previous incidence of preterm delivery [16], repeated surgeries of the abdomen or second trimester abortion [17], uterine and cervical limitations (growth retardation) [16], multiple pregnancy [18], *in-vitro* fertilization [12], smoking [16], lack of education and low socioeconomic status [19], diabetes before conception and chronic hypertension (preeclampsia) [20], and infection by asymptomatic bacteria [21]. Infection accounts for 30%–40% of early spontaneous preterm deliveries (26–28 weeks of gestation), and this is the same subset of preterm delivered babies who suffer from long-term morbidity and mortality [12]. Diagnostic tools such as biochemical markers of infection and hope for novel treatments have made “Infection and its inflammatory responses” prime attention of our study. 

## 2. Infection and Preterm Birth

Evidence from mice, rabbits, and rhesus monkeys shows that introduction of microbes or endotoxins into the pregnant animals induces preterm delivery [22, 23]. The ascending pathway is the most common route for intrauterine infection in humans [24]. Among the many suspected species of bacteria causing preterm delivery, *Ureaplasma urealyticum*, *Mycoplasma*, and *Fusobacterium* are most commonly isolated from amniotic cavities of women with preterm deliveries [24]. Watts and colleagues studied the amniotic fluid (AF) samples of women with intact membranes in idiopathic preterm labor [25]. Their studies establish an inverse relationship between the frequency of positive bacterial cultures and gestational age. Bacterial infection was most frequently observed in samples of women with labor at less than 30 weeks of gestation. Results suggest that women with positive cultures had a mean gestational age of 27.5 weeks. It should be noted that women with higher gestational age (>30 weeks) were less susceptible to intrauterine infection. Thus, infection usually occurs at early gestational age (<26 weeks). Neonatal respiratory problems, bronchopulmonary abnormalities, and death are consequences of intrauterine infection [25].

Gomez and colleagues have proposed that intrauterine infection-induced preterm delivery is a presentation of the basic phenomenon: activation of the host-defense macrophage system [26]. Preterm labor or preterm premature rupture of membranes (PPROM) results from the stimulation of the host response: initiation of uterotonic agents like prostaglandins (PGs) and the production of proteases (leukocyte elastase and matrix metalloproteinases) [26–29]. Research in animals and in human subjects has shown a cascade of biochemical events occurring as a result of intrauterine infection during gestation. In the event of intra-amniotic infection or choriodecidual space infection, inflammatory cytokines such as IL-1*α*, IL-1*β*, IL-6, IL-8, and granulocyte colony-stimulating factor are released [26–30]. Cytokines commence the synthesis of prostaglandins while neutrophil assisted chemotaxis promotes infiltration resulting in the release of matrix metalloproteinases (MMPs) and other bioactive substances [26–30]. Uterine contractions are initiated by PGs and members of the MMP family such as MMP9 and MMP2 which degrade structural collagens causing ripening of the cervix and rupture of the chorioamniotic membranes [31–35].

Intrauterine infection occurs at an early gestational age, but it is silent and asymptomatic. No signs of infection such as fever, blood leukocytosis, pain, or fetal distress are apparent until infection results in preterm labor [36]. Therefore, infection-associated markers prove to be the most useful tools to identify women with intrauterine infection. Predictors of infection-associated spontaneous preterm delivery include tumor necrosis factor-*α* (TNF-*α*), IL-6, IL-1, and IL-8. Although the aforementioned markers indicate the presence of intrauterine infection, vaginal/cervical fibronectin remains the prime predictor of spontaneous preterm delivery and is closely related to inflammation of fetal membranes followed by fetal sepsis [37, 38]. Oncofetal fibronectin belongs to the family of trophoblast proteins responsible for the attachment of the placenta to the uterus throughout gestation [39]. Inflammation-induced proteolysis may lower the affinity of oncofetal fibronectin for the uterine wall, facilitating placental-uterine detachment and release of the protein in vaginal secretions [40]. A positive test for oncofetal fibronectin in cervicovaginal secretions in the second or third trimester increases the risk of spontaneous preterm birth by 40–60-folds [37]. Discovery of new markers for preterm birth has not brought a significant decrease to the preterm birth rates. Therefore, novel treatments along with the signature markers may help us prevent or reduce the number of preterm deliveries and related mortality and morbidity. 

Tocolytics, agents inhibiting myometrial contractions, have been used as treatment for preterm delivery. Ritodrine was the only tocolytic agent approved by the Food and Drug Administration (FDA) [41]. Comparative studies among ritodrine and nifedipine or indomethacin show delayed delivery for at least 48 hrs. On the other hand, therapy had no improvement in maternal side effects or preterm adverse effects to the infant [42, 43]. Randomized trials for antibiotics like metronidazole or clindamycin have shown promising results in treating maternal intrauterine infection when used for prophylaxis. On the other hand, minimizing the incidence of infection has not been shown to decrease mortality, nenonatal sepsis or preterm birth rate [44]. While researching preterm birth, our prime intentions are to elucidate common pathways in the pathology of preterm delivery and minimize perinatal morbidity and mortality. 

## 3. Endothelins

Endothelin (ET) is a peptide which was known as endothelium-derived contracting factor (EDCF) until 1988 [45]. ET is the most potent vasoconstrictor peptide with slow onset and prolonged effect. It is a 21 amino acid peptide containing disulphide bonding among four cysteine residues as Cys^1^-Cys^15^ and Cys^3^-Cys^11^. These disulphide linkages are responsible for the high affinity of ET towards the ET_A_ receptor in comparison with the ET_B_ receptor. Studies of the human genome uncovered three different ET peptides: ET-1, ET-2, and ET-3. [45]. 

ET-1 regulates its effect by interacting with the ET_A_ receptors on the cell surface. ET-1 causes vascular contraction in response to a number of transduction mechanisms as follows: (i) facilitating Ca^2+^ influx, thereby increasing cytosolic free calcium concentration, (ii) stimulating phospholipase C, producing inositol 1,4,5-trisphosphate (IP_3_) and diacylgycerol (DAG), further activating PKC, and (iii) activating phospholipase A_2_ and arachidonic acid metabolism, catalyzing the first step towards inflammation [45]. The human endometrium has shown specific binding sites for ET-1 and ET-3, while the placenta produces a large quantity of big ET-1, which, once converted to ET-1, is the most potent molecule for contraction of human myometrium [46].

Romero et al. studied the concentrations of ET-1 and ET-2 in amniotic fluid samples of patients with full-term pregnancies with and without labor and patients with infection-associated and noninfection-associated preterm birth and samples from second trimester pregnancies [47]. Radioimmunoassay was used to definitively measure ET-1 and ET-2 [47]. Spontaneous labor at higher gestational age or full-term delivery did not show any changes in amniotic fluid concentrations of these peptides. ET-1 and ET-2 concentrations were increased, however, in samples with preterm labor and positive amniotic cultures when compared to those preterm labor samples without microbial infection [47]. These results suggest an important role of the endothelins in the pathological mechanisms leading to preterm birth in the presence of intra-amniotic infection. An *in vitro* study of ET-1 and ET-3 in comparison with oxytocin was carried out on samples of arteries and myometrial strips. ET-1 displayed powerful myometrial contractions in comparison to ET-3 and oxytocin. Taken together, these results suggest that endothelins have potent oxytocic effects [48].

Additional *in vitro* studies of uterine tissues show 75% increased contractile reaction and higher sensitivity to ET-1 at spontaneous delivery than the tissues from mid gestational age pregnant rats (day 18 of gestation). It is also observed that the reactivity for ET-1 in these tissues decreases on the first day postpartum and disappears completely by the second day postpartum [49]. Appropriate assay conditions of 22°C for 1–3 h were optimized to measure the ET-1 specific maximum binding sites. Using these assay conditions, ET-1 binding to ^125^I labeled ET-1 (^125^I-ET-1) sites was measured in these tissues. ET-1 displaced ^125^I-ET-1 in a dose dependent manner in comparison with ET-3, suggesting the presence of the ET_A_ receptor subtype [49]. The concentrations of ET-1 receptor are as follows: delivering animals expressed 280 ± 36 fmol/mg protein *n* = 4, whereas nondelivering animals expressed a concentration of 170 ± 30 fmol/mg protein, *n* = 4 [49]. These results clearly demonstrate a 1.7-folds increase in ET-1 binding sites in myometrium of animals delivering preterm compared to the concentration of the binding sites at term [49]. Breuiller-Fouché et al. report contradicting results demonstrating the effect of IL-1*β* on ET-1 concentration and its receptors [50]. A decrease in prepro-ET-1 and ET-3 mRNA was observed because of prolonged exposure to IL-1*β*. Instead, IL-1*β* overexposure failed to produce any effect on ET_A_ receptor expression but an unpredictable increase in ET_B_ receptors was observed. Researchers justify these paradoxical results stating that all the above-mentioned events may be taking place as regulatory mechanisms in response to opposing the onset of infection- associated preterm myometrial contractions [50]. HJP272, a 1,3,6-trisubstituted-2-carboxy-quinol-4-one, is a novel ET_A_ receptor antagonist, synthesized by our group, effective in preventing (low LPS dose) or postponing (high LPS dose) preterm delivery in animal models [51]. These lines of investigation suggest a significant role of ET in the regulation of preterm delivery in the presence of infection and a potential therapeutic role for ET antagonists. On the other hand, ET antagonists as tocolytics are a matter of concern, because the antagonists are considered Category X drugs. If administered as potential tocolytics; however, ET antagonists would be given transiently and after organogenesis was complete. Taking those factors into account and, moreover, considering additional potential advantages such as their effect on preeclampsia and intrauterine growth restriction, the benefits of ET antagonists in cases of preterm labor may outweigh the risks. Nevertheless, the concern about teratogenicity persists. 

## 4. Sphingosine Kinase

Sphingosine-1-phosphate (S1P) is a phosphorylated metabolite of sphingolipid, which has been highly conserved throughout evolution in yeasts, plants, and mammals [52]. In 1884, because of its enigmatic nature, sphingosine was named after the Greek mythological creature, Sphinx [53]. S1P was discovered to be an active regulator of cell proliferation, survival and, cell death. Moreover, S1P is highlighted as a signaling molecule governing vital biological responses in lower organisms such as plants, flies, slime mold, and yeast [54]. The riddle of such a simple molecule playing such a variety of roles is solved by the finding that it is a member of the family of lipid mediators that function as ligands (agonists) on specific cell surface receptors as well as signaling molecules inside the cell. The sphingosine kinase (SphK) enzyme catalyzes the ATP dependent phosphorylation of sphingosine into S1P. In mammals, two isozymes SphK1 and SphK2 have been identified [55]. S1P has been recently found to act as a natural ligand for the endothelial differentiation gene (EDG) family of G-protein coupled receptors (GPCRs) [56]. Five different members of the family exclusively binding to S1P and dihydro-S1P are as follows: EDG1/S1P1, EDG5/S1P2, EDG3/S1P3, EDG6/S1P4, and EDG8/S1P5. They are ubiquitously present to modulate diverse downstream signals. S1P receptors also participate in regulating GTPases like Rho and Rac [57], which are vital for cytoskeletal arrangement and chemotaxis or directed cell movement [58, 59]. All the above mentioned findings contribute to our knowledge about the ability of S1P to manage various physiological processes, including angiogenesis and tumor growth, heart development [60], and immune function [59] by specifically managing the relative expression of S1P receptors along with GPCRs.

 SphK/S1P participates during gestation and is now found to play a role in various processes such as quiescence, contraction, and apoptosis during pregnancy [61]. Cyclooxygenase (COX) exists as COX-1 and COX-2 isoforms. COX-2 is a crucial enzyme for the production of prostaglandins (PGs) in the uterus. Moreover, kinases such as PKC, MAPKs, ERK, and p38 play a major role in upregulating COX-2 [62]. Leiber et al. have recently discovered the link between S1P and ET-1. ET-1 induces SphK1 and Rho kinase through Ca^2+^ sensitization which comprises an important mechanism in normal parturition [63]. The evidence for SphK inducing production of COX-2 during parturition was uncovered by Serrano-Sanchez et al. [61]. They observed an elevation of SphK1/SphK2 at day 19 when pretreated with progesterone in rat myometrium whereas the effect was abolished postpartum. Previous studies suggest that SphK is activated by PKC, which in turn activates ERK in rat myometrial cells [63]. Their studies show S1P to be autocrine in nature in myometrial tissues, which in orchestration with SphK, PKC, and ERK, leads to the induction of COX-2, an abiding mechanism during labor [61]. 

The relationship between SphK and ET-1 mediated contraction is explained in the research of Leiber et al. [63]. With the use of phosphorylated FTY720 (which interacts with all S1P receptors except S1P_2_), they were unable to find any contractile response to S1P, confirming the fact that the S1P_2_ receptor is the one involved in this contraction. Furthermore, the contractile action of ET-1 was reduced by inhibition of SphK. Their research demonstrates a clear ET-1 mediated contraction pathway: PKC and PLC activation is upstream of SphK activation and Rho kinase activation leading to contraction is downstream [63].

Data generated by Tanfin et al. further support the previous studies mentioned. They used ELT3 uterine leiomyoma cells that released S1P synthesized by the enzyme SphK1 and not SphK2. This result was confirmed by using PDBu, which activated SphK1 only. The necessity of PKC and MAP kinase ERK1/2 was demonstrated when the release of S1P was inhibited by using Ro-318220 and BIM (PKC inhibitors), U0126 and PD98059 (MEK inhibitors), as well as SKI-II inhibitor and SphK1-siRNA [64]. Their studies suggest the role of an important molecule in this physiological pathway, ATP-binding cassette (ABC) transporter-ABCC1. Release of S1P was abolished by none of the inhibitors of ATP Binding Cassette transporters (ABCA1, ABCB1, and ABCC1) except ABCC1. Moreover, COX-2 expression was also blocked by inhibition of PKC, ERK1/2, SphK1, and transfected ABCC1-siRNA [64]. The SphK/S1P axis, acting downstream of ET-1 and dependent on ABCC1, represents an important junction in the putative pathogenetic mechanism of infection-triggered preterm labor and delivery.

## 5. Conclusion

**Figure 1:**
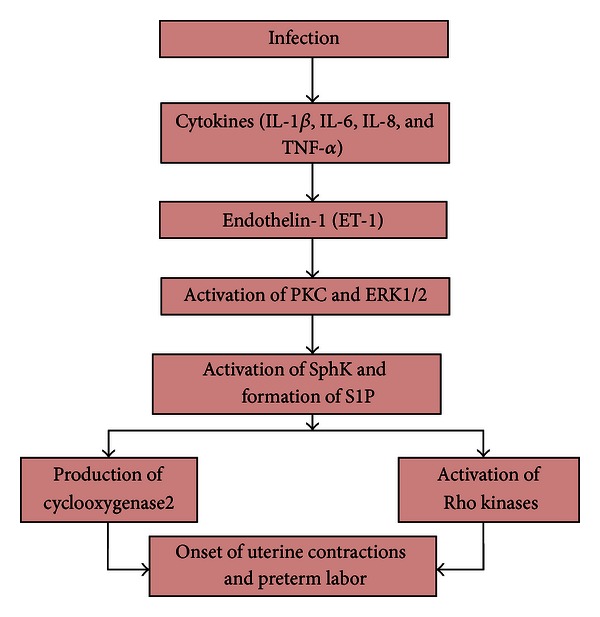
The Parturition cascade, showing the role of ET-1 and SphK.

 Intrauterine infection is always accompanied by an inflammatory reaction involving cytokines and a cascade of biochemical signals resulting in the onset of myometrial contractions and preterm delivery. Reports from various experiments suggest the parturition cascade involving ET-1 and SphK as shown in Figure 1. 

Although ET-1 is a significant proinflammatory mediator and smooth muscle constrictor and could be targeted in the development of novel pharmacotherapy to prevent preterm delivery, its Category X status may limit its use as a tocolytic. SphK is implicated as one of the members of the ET-1 induced parturition cascade and thus could be of use in the future as a treatment for preterm delivery in lieu of ET-1. Moreover, its role in the setting of preterm delivery still remains to be elucidated. Therefore, many mechanisms still remain to be discovered in order to accomplish effective treatment for this long-time challenge of preterm birth.
